# TLR4 Signaling Pathway Modulators as Potential Therapeutics in Inflammation and Sepsis

**DOI:** 10.3390/vaccines5040034

**Published:** 2017-10-04

**Authors:** Nikolay N. Kuzmich, Konstantin V. Sivak, Vladimir N. Chubarev, Yury B. Porozov, Tatiana N. Savateeva-Lyubimova, Francesco Peri

**Affiliations:** 1 Department of Drug Safety, Research Institute of Influenza, WHO National Influenza Centre of Russia, 15/17 Professor Popov St, Saint-Petersburg 197376, Russia; kvsivak@gmail.com (K.V.S.); drugs_safety@mail.ru (T.N.S.-L.); 2 Laboratory of Bioinformatics, Institute of Pharmacy and Translational medicine, I.M. Sechenov First Moscow State Medical University, 8-2 Trubetskaya St., Moscow 119991, Russia; yuri.porozov@gmail.com; 3 Department of Pharmacology, Institute of Pharmacy and Translational medicine, I.M. Sechenov First Moscow State Medical University, 8-2 Trubetskaya St., Moscow 119991, Russia; tchoubarov@mail.ru; 4 Laboratory of Bioinformatics, ITMO University, 49 Kronverkskiy Pr., Saint Petersburg 197101, Russia; 5 Department of Biotechnology and Biosciences, University of Milano-Bicocca, Piazza della Scienza 2, Milano 20126, Italy

**Keywords:** TLR4, sepsis, LPS, CD14, MD-2, in vivo studies, PAMP, DAMP

## Abstract

Toll-Like Receptor 4 (TLR4) signal pathway plays an important role in initiating the innate immune response and its activation by bacterial endotoxin is responsible for chronic and acute inflammatory disorders that are becoming more and more frequent in developed countries. Modulation of the TLR4 pathway is a potential strategy to specifically target these pathologies. Among the diseases caused by TLR4 abnormal activation by bacterial endotoxin, sepsis is the most dangerous one because it is a life-threatening acute system inflammatory condition that still lacks specific pharmacological treatment. Here, we review molecules at a preclinical or clinical phase of development, that are active in inhibiting the TLR4-MyD88 and TLR4-TRIF pathways in animal models. These are low-molecular weight compounds of natural and synthetic origin that can be considered leads for drug development. The results of in vivo studies in the sepsis model and the mechanisms of action of drug leads are presented and critically discussed, evidencing the differences in treatment results from rodents to humans.

## 1. Introduction

Toll-like Receptors (TLRs) are type I transmembrane proteins and are a panel of conserved pattern-recognition receptors (PRR) that are activated by a variety of pathogen-associated molecular patterns (PAMPs), thus initiating an innate immune response and inflammation in higher animals [1,2]. Toll-Like Receptor 4 (TLR4) is the member of TLR family that recognizes and is activated by bacterial lipopolysaccharide (LPS), which is the main molecular component of the cell wall of Gram-negative bacteria [3,4,5]. As other TLRs, TLR4 has a modular structure composed by a domain constituted by leucine-rich repeats (LRR) [6] in the extracellular part, connected to an intracellular TIR domain responsible for the signal transmission. Molecular recognition of minute amounts of circulating LPS (endotoxin) by the TLR4 receptor system, followed by receptor dimerization on the cell membrane, starts the cascade of protein-protein interactions leading to the production of pro-inflammatory cytokines and interferons, thus launching the inflammatory and immune responses.

### 1.1. The Extracellular TLR4 Receptor System

**Figure 1:**
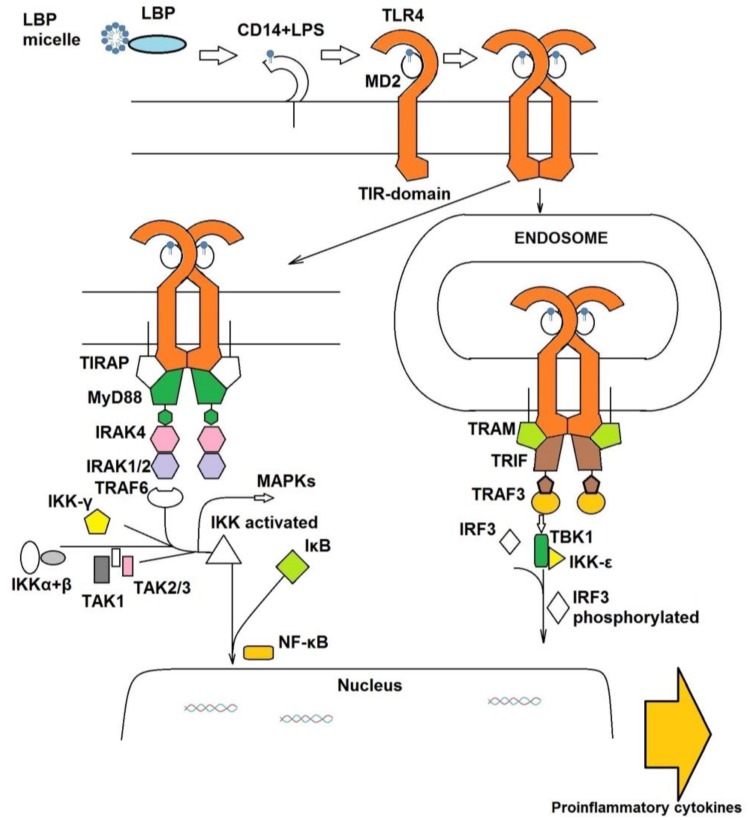
The lipopolysaccharide (LPS)/toll-Like Receptor 4 (TLR4) signaling pathway: the extracellular part (mediated by LPS-binding protein (LBP), cluster of differentiation 14 (CD14) and MD-2) and the intracellular part (myeloid differentiation primary response gene (MyD88) and TIR-domain-containing adapter-inducing interferon-β (TRIF) branches).

TLR4 does not bind LPS directly, and the adaptor protein MD-2 (also known as lymphocyte antigen 96 [7] is required, that directly binds and recognizes the lipophilic part of LPS (lipid A) forming a discrete complex [8,9]. It associates non-covalently to TLR4 to form the final activated heterodimer (LPS/MD-2/TLR4)_2_ [10] that in its turn starts the intracellular signal (Figure 1).

The dimerization of TLR4 is promoted by key interactions between MD-2, endotoxin’s lipid A sugars and fatty acid chains, and the two TLR4 (named TLR4 and TLR4’) on the extracellular side and by interactions of TIR domains (mainly TIR/TIR surfaces interactions) of the two TLR4 on the inner side [10]. TLR4 activation by LPS is accomplished through a series of sequential steps in which LPS is bound by different LPS-binding proteins and transferred to MD-2/TLR4 [11]. The LPS-binding protein (LBP) binds a LPS monomer from LPS aggregates in solution, transfers this molecule to cluster of differentiation 14 (CD14) protein, that finally chaperones the formation of the complex of LPS with MD-2/TLR4 [12]. CD14 is expressed mainly in macrophages and monocytes, as TLR4 it has leucine-rich repeats (LRR) and occurs in both soluble and membrane-bound (through a glycophosphatidylinositol (GPI) anchor) forms [13]. 

### 1.2. The Intracellular Signal Cascade

Once the sequential action of LBP and CD14 has promoted the formation of the activated TLR4/MD-2 heterodimer on the cell surface, the intracellular signal can follow one of two distinct directions, the TLR4/MyD88/NF-kB and TLR4/TRIF/IRF3 pathways (Figure 1). For further downstream signaling, the adaptor proteins MyD88 (myeloid differentiation primary response gene 88), TIRAP (TIR domain-containing adaptor protein), TRAM (TRIF-related adaptor molecule), and TRIF (TIR-domain-containing adapter-inducing interferon-β) are necessary [14]. The intracellular part of TLR4 and all of the TLR4 adaptor proteins possess Toll/interleukin-1 receptor (TIR) domains that are responsible of mutual interactions [14]. MyD88-dependent and TRIF-dependent pathways are competitive and mutually exclusive [15]. TLR4/MyD88 pathway starts from the (LPS/MD-2/TLR4)_2_ complex located on plasma membrane, whilst TLR4/TRIF transduction begins after complex internalization into endosomes. It is possible to dissect the two pathways by using molecules that selectively act on endocytosis. Shim et al. discovered that the anti-inflammatory action of some antimicrobial peptides is based on the inhibition of TLR4 endocytosis in LPS-stimulated cells that also blocks the TRIF-dependent branch of TLR4 signaling [16]. Interestingly, it has also been observed that CD14 plays a key role in promoting the internalization of TLR4-MD2-LPS complex into endosomes [17].

In the MyD88-dependent pathway, the TIRAP adaptor is recruited to the TIR-TIR dimer of the two TLR4 constituting the activated heterodimer, and the binding occurs via its TIR-domain. TIRAP is an intracellular protein and possesses a phosphatidylinositol 4,5-bisphosphate-binding domain that is necessary for membrane anchoring, and forms a homodimer [18,19]. It binds to the TIR-domains of TLR4 and together they form interface for arrival and joining of MyD88. 

MyD88 molecules form a complex and bind serine/threonine kinases, Interleukin-1 receptor-associated kinases 2 and 4 (IRAK2 and IRAK4), forming the so-called myddosome. The structure of the complex has been structurally resolved by X-ray crystallography [20]. It has been revealed that the stoichiometric ratio of MyD88-IRAK2-IRAK4 ensemble is 6:4:4. The myddosome formation promotes IRAK4 autophosphorilation [21]. IRAK1 can also bind to the MyD88-IRAK4 complex and be phosphorylated by IRAK4.

Next, TNF receptor associated factor 6 (TRAF6) is recruited, which forms a trimer [15]. It binds to phosphorylated IRAK1 and TRAF6, E3 ubiquitin ligase, promotes poly-ubiquitination of itself at Lys63 site. Polyubiquitin chains of TRAF6 are recognized by TAB2/TAB3 adaptor proteins and IKKγ subunit of IKK-complex [21]. It allows for the recruitment and activation of TAK1 and phosphorylation of IκB complex, respectively, promoting its degradation and release of NF-κB [22]. TAK1 activates various mitogen-activated protein kinases [23], and along with NF-κB they induce the production and release of proinflammatory cytokines IL-1β, TNF-α and IL-6 [24]. 

Analogously, binding the TRIF-related adaptor molecule (TRAM) to the intracellular TLR4-TIR domains is necessary for adaptor recruitment in the TLR4/IRF3 pathway [25]. Just as TIRAP, TRAM is a membrane-bound bridging adaptor [26] and forms a homo-oligomer [27]. The importance of TRIF for LPS response have been demonstrated in vivo, when TRIF-KO mice were protected from severe sepsis in the cecal ligation and puncture (CLP) model [28]. To activate and to recruit IRF3, phosphorylation of TRIF is required [29]. Phosphorylated IRF-3 then dimerizes and translocates to the nucleus to initiate the transcription of the IFN-β gene [30].

## 2. Pathologies Related to TLR4 Signaling

In addition to PAMPs, TLR4 can be also activated by damage-associated molecular patterns (DAMPs) derived from damaged and necrotic tissues (sterile inflammation), such as fibronectins, small fragments of hyaluronan, and even saturated fatty acids in response to cellular damage [31]. Besides the exogenous stimuli, endogenous host molecules, such as the oxydized phospholipids or high-mobility group box 1 (HMGB1) have also been shown to activate TLR4 [32,33]. While different LPS shares a conserved lipid A moiety with chemical determinants that ensure the optimal interaction with CD14 and MD-2 (5 or 6 lipophilic fatty acid chains attached to a disaccharide backbone, and one or two phosphate groups) DAMPs are chemically diverse molecules and the molecular mechanism of TLR4 activation including the role of CD14 and MD-2 in the sensing of these molecules are not entirely understood. DAMPs have been implicated in many pathologies caused by TLR4 activation, including atherosclerosis [34], rheumatoid arthritis [35], neuroinflammation [36], and trauma and hemorrhage [37]. Very recently, TLR4 has been suggested as a promising therapeutic target for drug abuse [38] and major depressive disorders [39,40] , as well as amyotrophic lateral sclerosis [41]. Possible application of TLR4 antagonists in treatment of peripheral neuropathic pain has also been discussed [42,43].

### 2.1. Sepsis and Septic Shock

Among PAMP/TLR4 diseases, sepsis is the most serious one. It is an excessive and dysregulated response of the host organism to outer pathogens, which leads to acute life-threatening organ dysfunction [44,45] . The global incidence of this syndrome accounts for 437 per 100,000 person-years between the years 1995 and 2015, according to retrospective analysis of an international database [46]. In western countries, mortality in patients with severe sepsis is 20­–50%, if there is no organ dysfunction it can be diminished (less than 20%) [47]. Septic shock with increased lipopolysaccharide (LPS) levels in blood, overexpression of pro-inflammatory cytokines, activation of blood coagulation system, and accumulation of fibrinogen degradation products leads to a violation of local and general hemodynamics and endothelial dysfunction via toll-like receptors signaling pathway [48].

Sepsis is also one of the possible complications of severe influenza. The most typical flora complicating disease is *Streptococcus pneumoniae*, *Pseudomonas aeruginosa*, *Acinetobacter spp*., *Staphylococcus aureus,* as well as *Enterobacteriaceae spp.* [49], *Aspergillus spp*. [50] and other.

Moreover, it has been recently discovered that the lethality of some influenza virus strains (human pandemic H1N1 or PR8) is due to abnormal TLR4 activation by endogenous factors (DAMPs), such as oxidized phospholipids, generated as a consequence of the acute lung injury (ALI) caused by the viral infection [51,52].

Because of TLR4 signaling cascade’s huge role, its extracellular and intracellular components are very attractive therapeutic targets for the treatment of both acute (e. g., sepsis) and chronic disorders, associated with excessive cytokine production (also called, in the case of sepsis, cytokine storm) [53,54,55,56,57,58].

### 2.2. Animal Models of Sepsis

Rodents have been widely used as animals for studying sepsis. Several models have been developed so far—LPS treatment, administration of viable pathogens, and caecal ligation and puncture model (CLP) [59,60,61,62]. In the latter case the endogenous protective barrier is damaged and pathogen efflux follows.

LPS injection model is easy to perform and the induced inflammatory response has a good reproducibility. High levels of pro-inflammatory cytokines are released soon and systemic inflammatory response syndrome (SIRS) develops rapidly followed by dose-dependent mortality. The disadvantage of this method is that pathophysiological aspects of human sepsis are not fully reproduced [60]. Bacterial injection model (also known as peritoneal contamination and infection model, PCI) is better since it mimics microbial sepsis and especially the polymicrobial one, which cannot be induced by endotoxin administration although extensive bacteremia is rarely observed by sepsis patients [63].

CLP model is the most widely used one and is considered to describe best the human sepsis. The bacterial endotoxin release into the bloodstream is relatively slow and can be adjusted by the number and size of punctures [64,65]. With respect to the IL-6 and TNF-α, hemodynamic, and biochemical responses CLP model is the most comparable to human sepsis [66,67].

Various compounds have been tested on animal models for their capacity to block TLR4-mediated cytokine production, and several have reached the clinical trials. The known TLR4 antagonists belong to various classes of chemical compounds—mainly glycolipids that mimic the natural TLR4 ligand, lipid A, but also heterocycles, peptides, opioids, taxanes, steroids, etc, and have natural and synthetic origin.

## 3. TLR4 Antagonists from Natural Sources

Plant secondary metabolism provides a vast source of chemically diverse bioactive and pharmacologically active compounds. Traditional Chinese and Indian medicine use a variety of herbs that are rich in molecules that very likely act as TLR4 modulators. TLR4 activation or inhibition mediated by herbal extracts promoted a vast area of research that focuses on the molecular mechanism of action of these TLR4 modulators.

**Figure 2:**
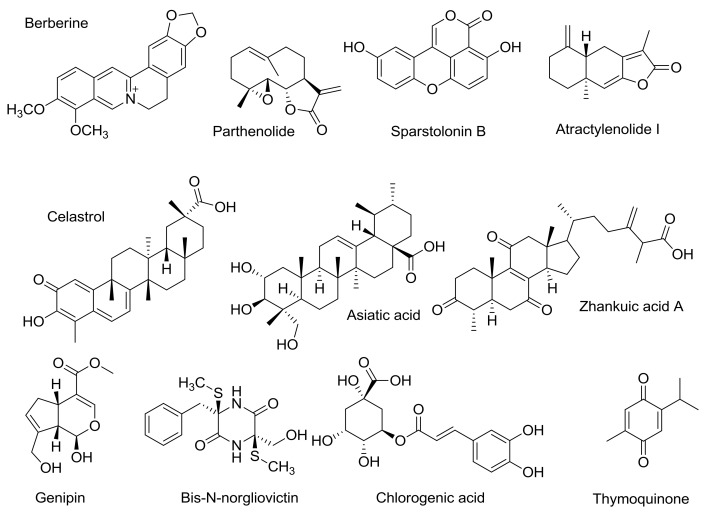
Natural compounds with TLR4-antagonistic properties, tested *in vivo* on animal models of sepsis.

Berberine (Figure 2), an isoquinoline alkaloid mainly extracted from *Rhizoma Coptidis*, significantly postponed the death after intraperitoneal LPS (from Salmonella thyphimurium LT2) injection in mice, decreased the body temperature on LPS-generated fever in rabbits, and inhibited the increasing of nuclear factor kappa-light-chain-enhancer of activated B cells (NF-κB), interleukin-6 (IL-6), tumor necrosis factor alpha (TNF-α), and interferon-beta (IFN-β) expressions (real-time PCR analysis of mRNA expression) [68]. Docking studies were done by using Autodock 4.2 software [69,70]. AutoDock 4 is based on free energy force field parameterized using a large number of protein inhibitor complexes. For both inhibition constants (Ki) and structures are known. Docking analysis suggested that berberine can bind to MD-2 and that the MD-2 hydrophobic binding pocket is able to accommodate up to three berberine molecules [68]. Besides binding to MD-2, berberine also blocks TLR4/NF-κB transduction at a later stage directly binding the cysteine 179 residue of IκB kinase (IKK), and thus suppressing NF-κB activation through the inhibition of phosphorylation and degradation of IκBα [71]. Because its dual targeting (MD-2, extracellular and IKK, intracellular), berberine can be considered a promising hit to develop drugs that efficiently block the LPS/TLR4 signaling at different points.

Parthenolide (Figure 2) is a known inhibitor of the TLR4/NF-κB pathway [72]. It has been observed in human leukemia monocytic THP-1 cells that the LPS-stimulated production of TNF-α, as well as the production of various interleukins (IL-6, IL-1β, IL-8, IL-12p40, IL-18), were reduced more than 50% by the administrating parthenolide. Moreover, parthenolide was active in reducing levels of TLR4 expression after LPS activation. Similar results were obtained on human keratinocytes [73]. Biochemical studies suggest that this sesquiterpene lactone blocks both the MyD88- and TRIF branches of TLR4 signal pathway [74,75]. However, in vivo studies performed on different murine strains led to ambiguous results. In the LPS-induced septic shock model on Swiss albino rats, the administration of parthenolide improved survival [76]. On the contrary, parthenolide failed to improve and even deteriorated survival on C57BL/6J mice [77] on the same model of LPS-induced septic shock. The mechanism of action of parthenolide has been investigated by means of computational studies (AutoDock4) and it has been proposed that the TLR4 antagonism is due to parthenolide binding to TNF receptor associated factor 6 (TRAF6) [78].

Sparstolonin B (SsnB) isolated from a Chinese herb (*Sparganium stoloniferum*) (Figure 2), was found to significantly inhibit the expression of the cytokines TNF-α, IL-6, and IL-1β induced by LPS (mRNA concentrations of cytokines were measured by quantitative real-time PCR).

Treatment of macrophages with SsnB and LPS also caused several-fold decrease in TNFα and IL-6 levels, if compared with group treated by LPS only [79]. In this case, cytokine concentrations were measured by ELISA. In addition, SsnB attenuates TLR4-mediated NF-κB activation in a dose-depending manner and inhibits myeloid differentiation primary response gene 88 (MyD88) recruitment to TLR4 and suppresses LPS-provoked inflammation in mice (decrease in TNF-α and interleukin 1 beta (IL-1β) expression was statistically significant) [80]. Another study has demonstrated that SsnB increased the survival rate (4-fold) after intraperitoneal LPS administration both treating with SsnB before and after LPS administration. Additionally, pretreatment with SsnB significantly alleviated the lung pathology caused by LPS-injection. The latter two experiments were performed on mice [80]. Inhibition of LPS-induced inflammation in 3T3-L1 adipocytes [81] and human umbilical vein endothelial cells was also observed [82].

Atractylenolide I (Figure 2), a bioactive component of *Rhizoma Atractylodis macrocephalae*, significantly decreased LPS-induced TNF-α, IL-6, nuclear NF-kB p65 factor, extracellular signal–regulated kinases (ERK) 1 and 2, and p38 production by murine macrophage-like RAW264.7 cells [83]. Expression levels of MD-2, CD14, complement receptor 3 (CR3), SR-A, TLR4, and MyD88 were also significantly attenuated, as shown by Western blot analysis. This sesquiterpenoid has also proven to be effective *in vivo*. It protects mice from acute lung injury (ALI) induced by LPS [84] and significantly increased the survival rate on sepsis induced by CLP [85]. In this context, one can speculate that atractylenolide is active in antagonizing DAMP/TLR4 signaling. The normalization of liver and kidney functions as well as a significant decrease in serum cytokine levels was also observed. According to the docking studies, the possible mechanism of action for artactylenolide I involves binding to MD2 protein and preventing its interaction with LPS or DAMPs [86].

Zhankuic acid A (ZAA, Figure 2), isolated from the mushroom *Taiwanofungus camphoratus,* which is highly valued in Chinese traditional medicine, is a triterpenoid with a steroid structure. ZAA significantly blocks LPS-induced phosphorylation of ERK, c-Jun N-terminal kinase (JNK), p38, AKT, as well as NF-κBp65 phosphorylation, thus blocking NF-kB, mitogen-activated protein kinase (MAPK), and AKT signaling pathways. LPS- and *Salmonella choleraesuis* – induced TNF-α and IL-6 in vivo and in vitro production in RAW264.7 cells were both attenuated [87]. At a dose of 10 mg/kg (C3H mice, i.p.), ZAA was active in prolonging survival after LPS administration at the LD50 concentration (100% increase, *p* < 0.001). In the same conditions, 2 mg/kg of ZAA provided a 30% increase in survival as compared to control mice treated with LPS only. However, this variation is not statistically significant.

Docking studies (Dock 5.1 software [88]) proposed that ZAA can interact with the hydrophobic binding pocket of MD-2, that accommodates the lipophilic chains of lipid A, the natural MD-2 ligand. Dock 5.1 employs incremental construction for ligand sampling, merged target structure ensemble for receptor sampling, force-field based scoring function and distance dependent dielectric, generalized Born, and linearized Poisson-Boltzmann models. Consensus scoring analysis performed using the XScore scoring function [89] after generating binding pose predicted pK_d_ value of ZAA as high as 7.83, being two orders of magnitude higher than the reference substance LPS itself (pK_d_ = 5.83). However, no experimental data supporting direct binding of ZAA to MD-2 have been reported so far.

The triterpenoids celastrol and asiatic acid (Figure 2) are also active in disrupting TLR4 signaling. Experimental binding studies showed that celastrol binds non-covalently to MD-2 and then the interaction evolves in a covalent binding through Michael addition of celastrol to a thiol group of an MD-2 cysteine [90]. Both in vitro and in silico studies showed that celastrol compete with LPS for MD-2 binding [91]. Asiatic acid significantly diminished LPS-induced lung injury by male BALB/c mice in a dose-dependent manner [92]. Several other triterpenoids also exhibited IKKβ mediated activation [93].

Inhibition of both MyD88- and TRIF-dependent branches of TLR4-signaling was also observed by genipin, an aglycon of geniposide [94] and bis-N-norgliovictin, isolated from a marine fungus [95] (Figure 2). Genipin improved the survival of male ICR mice in both endotoxemia and CLP sepsis. The study of Kim and coworkers showed that attenuation of apoptotic depletion of T lymphocytes also contributes to the better survival in sepsis [96]. Bis-N-norgliovictin also improved survival after LPS administration, decreased serum cytokine levels and reduced lungs, and liver damage.

Chlorogenic acid (CGA) (Figure 2) is a major component of *lonicerae flos* extract. Intravenous administration of CGA protected C57BL/6 mice from septic shock after intraperitoneal LPS challenge [97]. At the dosage 3 mg/kg (CGA), the survival rate was increased up to 70%. In addition, the cytokine levels in blood of treated animals were decreased, too. In vitro, kinase assays demonstrated that MAPK activation was blocked by CGA, as well as auto-phosphorylation of IRAK4. Protein or mRNA levels of TNF-α, IL-1α, and HMGB-1 (high-mobility group box-1) in the peritoneal macrophages, induced by LPS, were also attenuated by CGA treatment.

*Lonicerae flos* extract (HS-23) itself has demonstrated similar results [98]. Apart from CGA, the extract also contains its isomers, cryptochlorogenic, and neochlorogenic acids, and also glycosides loganin and vogeloside. Loganin was found to inhibit NF-κB activation [99]. Moreover, HS-23 recently underwent stages I and II of clinical trials [98].

Thymoquinone (Figure 2) was proven to inhibit another interleukin-1 receptor-associated kinase, namely IRAK1 [100]. Preventive administration of thymoquinone significantly improved survival of albino mice after both *E.coli* and *E.coli*–derived LPS challenge (*p* < 0.01, log-rank test) [101]. Later, this group showed that organ dysfuction accompanying sepsis was diminished after treatment by this quinone [102]. The therapeutic potential of IRAK4 blocking has been outlined by Li [103]. Several reviews focused on IRAK4-inhibitors and their possible applications of inflammation and oncology disorders have been published recently [104,105,106,107,108].

**Figure 3:**
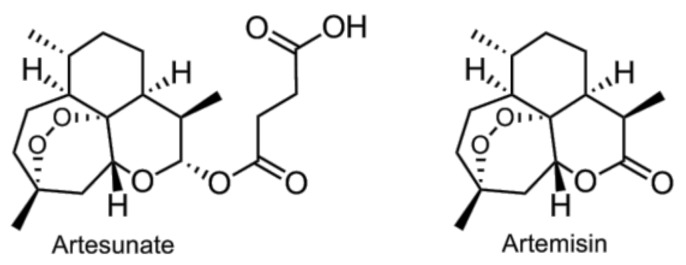
Artesunate and artemisinin.

Artesunate (AS) (Figure 3), a hemisuccinate derivative of dihydroartemisinin soluble in water, promoted to the decrease of TNF-α and IL6 levels in mouse peritoneal macrophages induced LPS, or heat-killed *E. coli* [109]. Pretreatment of Kunming mice with AS significantly decreased mortality and delayed the time of death. Endotoxin and TNF-α levels were also decreased dose-dependently. The suppression of TLR4/MyD88/NF-κB signaling pathway by AS was also observed in murine BV2 microglial cells [110]. The natural compound artemisinin (Figure 3), a precursor of AS, have also demonstrated a similar activity in vivo [111].

**Figure 4:**
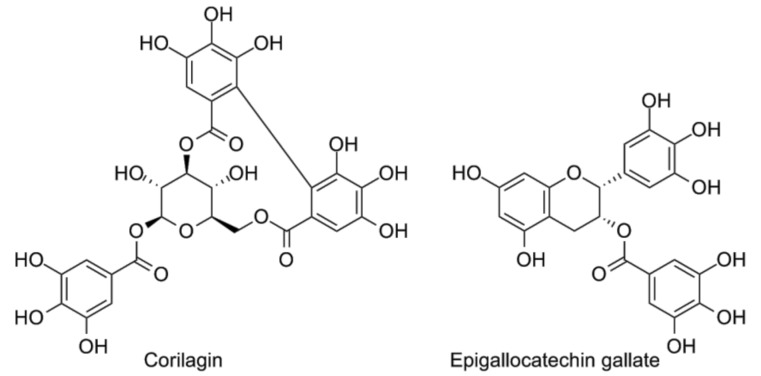
The polyphenols corilagin (left) and epigallocatechin gallate (right).

Recently, it has been discovered by Li et al. [112] that corilagin (Figure 4), which belongs to the group of hydrolysable tannins, also attenuates system inflammation in vivo after LPS injection. When administrated at a dose of 40 mg/kg, corilagin significantly decreased LPS-iduced lethality of Balb/c mice. In liver tissue the expression of TLR4, MyD88, TRIF, and TRAF6 proteins was increased after LPS injection also in animals treated with corilagin, but to a lower extent than in control animals treated with only LPS. In mouse serum, the same pattern of changes for IL-6, IL-1β levels was also observed. Epigallocatechin-3-gallate (EGCG) (Figure 4) is the most abundant polyphenolic flavonoid contained in green tea. Singh and co-workers [113] discovered that EGCG blocked TLR4/NF-kB pathway and selectively inhibited phosphorylation of TAK1 at the Thr184/187 site leading to a loss of its kinase activity. Inhibiting K63 auto-ubiquitination of TRAF6 was also observed. In experimental sepsis EGCG e treatment significantly improved both the hypotension in Sprague-Dawley rat and the survival of C57BL6 mice [114].

## 4. Synthetic TLR4 Antagonists

### 4.1. TAK-242 and Eritoran: Clinical Trials

**Figure 5:**
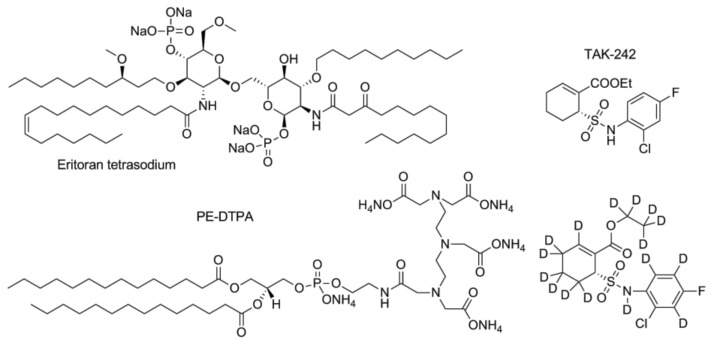
Eritoran tetrasodium, PE-DTPA, TAK-242 (resatorvid) and its deuterated analogue.

Several TLR4 antagonists were synthesized, the majority of them being mimetics of lipid A, the natural MD-2 ligand [115]. Lipid A is a glucosamine disaccharide with two phosphate groups in C1 and C4’ positions and six fatty acid chains. The binding of lipid A to MD-2/TLR4 is driven by the hydrophobic interaction of the fatty acid chains of lipid A with the MD-2 hydrophobic binding cavity, as well as polar interactions of the disaccharide backbone and phosphates with MD-2 residues at the rim of the cavity [10]. The most famous lipid A mimetic is Eisai’s Eritoran (Figure 5) that entered clinical phase. Other TLR4 antagonists with a chemical structure unrelated to lipid A have been recently developed. However, only TAK-242 (resatorvid, Figure 5) entered clinical trials.

TAK-242 (resatorvid) (Figure 5) is a small-molecule compound that selectively inhibits TLR4 signaling. TAK-242 inhibits the TLR4 pathway by binding directly to a Cys747 in the intracellular TLR4 domain [116] . It has been observed that TAK-242 disrupts the interactions of TLR4 with its adaptor molecules, TIRAP (toll-interleukin 1 receptor (TIR) domain containing adaptor protein), and TRAM (TIR domain-containing adapter inducing IFN-β-related adapter molecule). Treatment of the HEK293 cells transfected with plasmids encoding FLAG-TLR4, MD-2, and FLAG-TIRAP/FLAG-TRAM proteins with TAK-242 inhibited the co-precipitation of TIRAP with TLR4 in a concentration-dependent manner. TAK-242 inhibited the association of TRAM with TLR4 at concentrations similar to those at which it inhibited the association of TIRAP with TLR4. Another study also confirmed this mechanism of action [117].

Several other studies demonstrated the efficiency of TAK-242 on the murine sepsis model at both single and combined therapies [118,119]. Apart from the above, TAK-242 also protects against acute cerebral ischemia/reperfusion injury in mice by dose of 3 mg/kg [120]. On the acute kidney injury model (sheep, i.v.) there was a protective effect observed. The pathologic condition was induced by *E. coli*-derived LPS [121]. Several studies demonstrated TAK-242 anti-inflammation activity in other rodent species than mice [122,123]. Eventually, TAK-242 was admitted to the clinical trials. In a randomized, double-blind, placebo-controlled trial, embracing 274 patients with severe sepsis and shock or respiratory failure, there were two dose groups receiving TAK-242 1.2 and 2.4 mg/kg/day, respectively, and one group receiving the placebo. TAK-242 failed to suppress the interleukin-6 level even at a high dose group (*p* = 0.15). Organ-dysfunction assessments did not reveal any differences between placebo and treated groups. Finally, 28-day survival did not differ significantly between the treatment groups (*p* = 0.46, log-rank test) [124].

Fully deuterated TAK-242 (Figure 5) retains TLR4-antagonistic activity, while having better pharmacokinetic and distribution properties than TAK-242 [125].

Eritoran is probably the most known antagonist of TLR4. It mimics the lipid A, but presents four instead of six fatty acid chains, one of them being unsaturated. The crystallographic analysis of the Eritoran/MD-2 complex revealed that Eritoran binds MD-2 more similarly than lipid A, by accommodating the four fatty acid chains into MD-2 binding pocket [126]. However, when bound to MD-2 cavity, Eritoran is rotated 180° respect to lipid A [126]. According to this model, Eritoran acts thus as a classic competitive inhibitor of MD-2 competing with LPS for the binding of the MD-2 pocket. After successful results were obtained on animal models, Eritoran was suggested for testing on humans [127,128,129]. The pharmacodynamics study showed Eritoran to be safe and well-tolerated [130]. Later, at the phase 2 of clinical trials, Eritoran failed to diminish mortality rate even at a high dose 105mg (compared with placebo group, *p* = 0.335). The study was performed as prospective, randomized, double-blind, placebo-controlled, multicentre one [131]. Unfortunately, another randomized, double-blind, placebo-controlled, multinational phase 3 trial in 197 intensive care units did not show the optimistic results either. The all-cause mortality was not reduced for the primary (28 days) and secondary (1 year) end-points [132].

Results of clinical studies for TLR4-signaling blockers are summarized in Table 1. Phase 1 clinical trials of Eritoran are summarized in the review [128].

### 4.2. Synthetic Cationic and Anionic Amphiphiles

A synthetic phospholipid analogue, the cationic amphiphile 1,2-dimyristoyl-sn-glycero-3-phosphoethanolamine-N-diethyle-netriaminepentaacetic acid (also known as PE-DTPA, Figure 5), improved the survival of LPS-treated C57BL/6 mice in a dose-dependent manner, as compared with group of animals given LPS alone [133].

**Figure 6:**
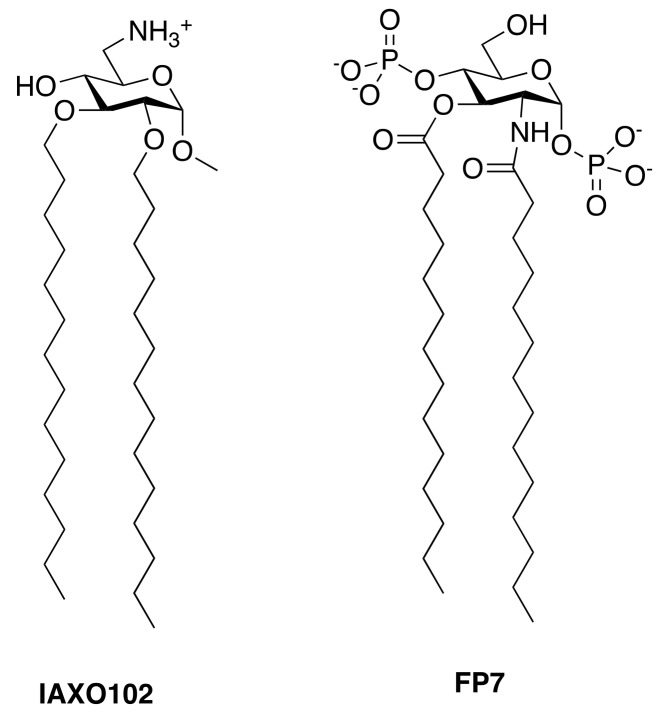
Positively and negatively charged monosaccharides active as TLR4 antagonists

Other cationic amphiphiles (IAXO compounds, Figure 6) based on monosaccharide scaffolds efficiently inhibited TLR4 signaling in vitro and in vivo [134].

The mechanism of the antagonist action of this class of compounds was studied in the case of IAXO-102 (Figure 6). A direct interaction of hydrophobic fatty acid chains of this compound and MD-2 was found by NMR measurements [135], confirming that very likely these compounds directly compete with LPS for MD-2 binding. Moreover, it has been observed in in vitro binding tests high affinity of IAXO compounds for CD14 [135], so that interaction with CD14 probably reinforces the antagonist effect on the TLR4 signal pathway.

Based on the success of IAXO compounds in inhibiting TLR4 signaling, other cationic amphiphiles were developed as TLR4 antagonists, as, for instance, trehalose derivatives [136]. 

Other negatively charged, monosaccharide-based TLR4 antagonists have been recently developed. Compound FP7 (Figure 6) inhibited the LPS-triggered, TLR4-mediated cytokine production in cells [137], and was found to be very active to contrast in vivo the TLR4-mediated lethality associated to influenza virus infection [52]. The mechanism of action of FP7, as in the case of IAXO compounds, is based on a combination of a direct competition with LPS for MD-2 binding and interaction with CD14. It was observed that, upon administration of FP7, CD14 endocytosis is stimulated so that after a certain time no CD14 is present on the plasma membrane [137]. The selective deprivation of CD14 from the cellular membrane is a peculiar mechanism adopted by this type of monosaccharide antagonists to inhibit TLR4 activation and signaling.

### 4.3. Chalcone Derivatives and Curcumin Analogues

**Figure 7:**
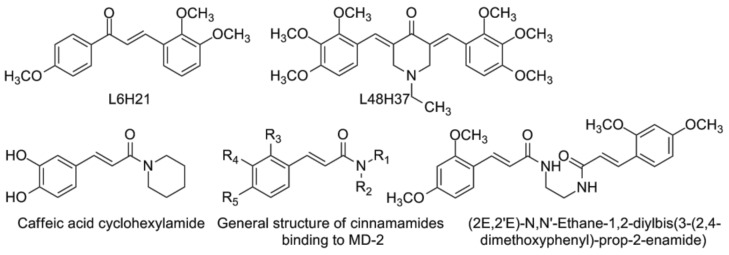
Chalcone L6H21, curcumin analogue L48H37 (upper row, from left to right), caffeic acid cyclohexylamide, general structure of synthesized cinnamamides, and the most active compound (from left to right, lower row).

A series of structurally related compounds (Figure 7) sharing the cynnamoyl fragment are known as MD-2 binders. Trimethoxychalcone L6H21 improved the survival of C57BL/6 mice after LPS administration as compared to untreated animals [138]. Binding to MD-2 was confirmed by surface plasmon resonance (SPR). Similar chalcones were effective in the LPS-induced acute lung injury model [139]. L48H37 was rationally designed as a curcumin analogue (Figure 7): it is stable under physiological conditions and binds to MD-2 [140], as confirmed by fluorescence spectroscopy and surface plasmon resonance (SPR) assays. Male C57BL/6 mice were injected with 200 μL of LPS (at 20 mg/kg i.v.) 15 minutes before (for treatment) or after (for prevention) the administration of L48H37 (10 mg/kg i.v.). After seven days, both treatment and prevention groups have shown significantly better survival (*p* < 0.01) than LPS-injected animals. Curcumin itself has proven effective on TLR4-signaling [141,142,143]. The recent meta-analysis of randomized controlled trials (609 subjects overall) has shown that curcumin consumption significantly decreases IL-6 plasma levels, especially by system inflammation [144].

Chen et al. [145] have prepared the series of various cinnamamides (Figure 7). Just as previously mentioned L48H37, they also have cinnamoyl fragment and bind to MD-2 as well. More than 30 compounds have been synthesized and tested in vitro on their ability to supress TNF-α production by mouse peritoneal macrophages after LPS stimulation. (2E,2'E)-N,N'-Ethane-1,2-diylbis(3-(2,4-dimethoxyphenyl)-prop-2-enamide) had been chosen among the four most promising structures and investigated in more detail. In C57 mice, the protection against LPS-injection induced sepsis (intraperitoneally) was observed and the survival rate was significantly increased. Caffeic acid cyclohexylamide (Figure 7, lower row, left) also belongs to the cynnamamides but in contrary to aforementioned compounds do not bind to MD-2. It was shown to inhibit IKKβ-kinase activity [146] and consequently the transcriptional activity of NF-κB. This caffeic acid derivative also rescued C57BL/6J mice after LPS-injection or CLP-induced sepsis, the group treated with 100 mg/kg dose showed 80% survival rate.

### 4.4. Other Compounds

**Figure 8:**
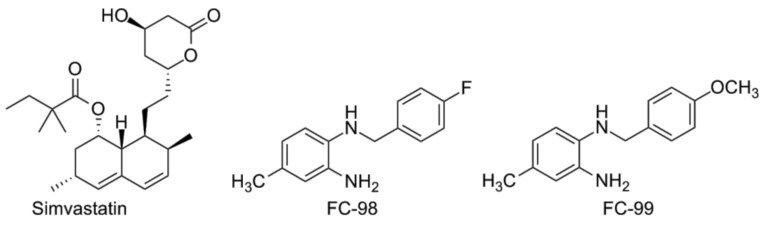
Simvastatin, FC-98 and FC-99 (from left to right).

In a double-blind, placebo-controlled study performed on male subjects (humans), simvastatin (Figure 8) caused statistically significant attenuation of LPS-induced TLR4 up-regulation in monocytes [147]. The substance was administered orally 80 mg a day. Also a decrease of TNF-α and MCP-1 plasma levels after LPS administration was noted.

In addition to the natural IRAK4 inhibitors mentioned above, the synthetic benzenediamine FC-99 (Figure 8) was proven to bind to IRAK4 both in silico and in vitro (SPR assay) [148]. FC-99 protected mice in the CLP-induced polymicrobial sepsis model and significantly decreased the serum levels of TNF-α and IL-6. A quite similar benzenediamine FC-98 also improved survival before as well as after LPS challenge [149]. In vitro blocking of both TLR4/NF-κB and TLR4/IRF3 pathways was observed. This finding is consistent with the existence of TRAF3-meditates bypass from IRAK1 to IRF3 [150].

Other important classes of natural and synthetic TLR4 antagonists have been reviewed recently by us and others (ref [115]). New TLR4 antagonists have been discovered in last two years, including synthetic compounds, such as fatty acid esters of monogalactosyl-diacylglycerol, trimannoside glycolipid conjugates (MGC), lipid A mimetic with Vizantin-like branched chains, morphine derivatives, and natural compounds as platycodin D, ferulic acid, chalcones, etc. and will be reviewed by us elsewhere (manuscript in preparation).

### 4.5. In Silico Studies

Compounds with TLR4-antagonstic activity were identified in silico among 100 structures sharing similarity greater than 70% to Eritoran, a known TLR4 antagonist, in the library consisting of 124413264 compounds. Testing on tissue obtained from Swiss Webster (CFW) mice has demonstrated that these substances inhibited LPS-induced NF-kB activation [151]. Pre-treatment with any of aforementioned compounds prior to LPS administration led to less severe symptoms of septic shock. Treatment with isopropyl 3,4,6-tri-O-acetyl-2-(acetylamino)-2-deoxyhexopyranoside, C = 10 μM, reduced TNFα mRNA levels in human necrotizing enterocolitis tissue. IL-6 levels were also attenuated after LPS-stimulation as determined by ELISA method (C3H/WT cells) [151]. In silico approaches to design and discovery of new TLR4 modulators are discussed in a recent review [152]. Several other reviews are also devoted to the computational approaches to the discovery of the new TLR4-modulators [153,154,155].

## 5. Peptide TLR4 Modulators

We focus now on antagonists of TLR-4 signaling which have a peptide structure. As we discussed above, for the TLR4-mediated signal transduction multiple protein-protein interactions are necessary. A valuable strategy for TLR4 antagonism would therefore be the use of short peptides that mimic protein epitopes and disrupt interactions of TLR4 with MD-2 or other adaptor proteins containing TIR domains.

### 5.1. Peptides that Disrupt the TLR4/MD-2 Interaction

A 17-residue peptide was projected in silico as a mimic of TLR4-binding region of human MD-2 (hMD-2) and synthesized [156]. This peptide (sequence: CRGSDDDYSFCRALKGE) bound to TLR4 with higher affinity than hMD2 (ΔG = −7.8 kcal/mol vs −5.5 kcal/mol).

Han et al. [157] have constructed decoy protein, which comprised the structure motifs necessary for interaction with MD2 and thus a competitive inhibitor was obtained. The mutation experiments led to the decoy receptor variants possessing higher affinity to MD-2.

### 5.2. Peptides that Disrupt TIR/TIR Interactions

TIRAP decoy peptides were designed and synthesized to disrupt the interaction of the TLR4-TIR domain with adapter proteins necessary for the downstream signaling [158]. It has been known that both TLRs and their adapter proteins have TIR domain that mediates the interactions necessary for the signal transduction [159,160,161]. TIRAP peptides blocked both MyD88-dependent and MyD88-independent cytokine genes induced by LPS, and consisted of 10–14 amino acid residues. Among them, five peptides were found to inhibit cytokine gene expression as well as MAPK after LPS stimulation [158]. In vivo, IL-6 and TNF-α concentrations were also attenuated in murine blood by the two most active peptides after administration of a sublethal LPS dose.

The same research group has designed and prepared TRAM adapter TIR domain derived decoy proteins applying the same approach [162]. Two of eleven peptides, IVFAEMPCGRLHLQ and ENFLRDTWCNFQFY, were the most potent to inhibit expression and secretion of all cytokines considered (TNF-α, IL-1β, IL-6, RANTES, IFN-β) in vitro. The activation of MAPKs was also inhibited. Co-immunoprecipitation assays have shown that these peptides block adapter recruitment to TLR4. Six truncated variants of IVFAEMPCGRLHLQ were also synthesized. The pentapeptide IVFAE was the most active among them and more potent than precursor with longer sequence. When administrated to the C57BL/6J mice, IVFAEMPCGRLHLQ, ENFLRDTWCNFQFY, and IVFAEMPCG effectively suppressed LPS-induced cytokine induction and protected animals from lethal endotoxemia after sublethal LPS dose. Treatment of mice with 10 nmol/g of peptide before the injection of LPS on the LD100 level either rescued all the animals (IVFAEMPCGRLHLQ and IVFAEMPCG) or at least part of them (77%, ENFLRDTWCNFQFY). Reversing the order of administration of peptides and LPS similar results were obtained.

Another way to block TLR4 signaling is to inhibit the dimerization of TLR4 TIR domains, which occurs by the stimulation of agonist and is necessary for downstream signal transduction. 12 peptides (each was 9–14 amino acids long) reproducing different parts of TIR-domain were synthesized and tested in vitro on murine macrophages stimulated by LPS. Five of them were the most active ones to reduce cytokines expression (IL-1β, TNF-α, IFN-β, and RANTES mRNA). Among these five, three (LHYRDFIPGVAIAA, AGCKKYSRGESIYD, and HIFWRRLKNALLD) demonstrated the best affinity to TLR4-TIR as have been shown by time-resolved fluorescence spectroscopy [163]. The same group also has designed and synthesized TRIF TIR domain-derived decoy peptides [164]. They also were effective in vivo, decreasing LPS-induced cytokine response and improving survival of mice after LPS challenge.

Decoy peptides designed to reproduce the binding part of TIR-domain of TcpB/Btp1 protein from *Brucella spp.* were also tested as blockers of protein-protein interactions necessary for TLR4 signal transduction [165]. The assumption was that TcpB/Btp1 can interact with adaptors downstream of TLRs, prevent NF-κB activation, and consequently diminish cytokine production, which is important for innate antibacterial immune response [166]. In vitro, two of the twelve synthesized peptides significantly inhibited mRNA expression of both MyD88-dependent (TNF-α and IL-1β) or TRIF-dependent (IFN-β) cytokines as well as their secretion. In Balb/c mice, pre-treatment with peptides significantly decreased cytokine levels after LPS administration.

It is worth to note that TIR homologue proteins occur in other bacterial species, including *Salmonella enterica* [167], *Staphylococcus aureus* [168]. Such proteins can modulate the host immune response [169]. Bacterial proteins containing TIR-domains are discussed in review by Rana et al. [170].

One more TRAM-derived peptide inhibits the inflammatory response in mouse mammary epithelial cells and a mastitis model in mice [171]. Hines et al. [172] have prepared blood-brain barrier (BBB)-permeating peptides, 18 amino acids long, to inhibit TLR4-MyD88 interaction. At concentration 3 μM, TNF-α production in the murine brain tissue after LPS stimulation was significantly decreased (ex vivo). Prior to peptide injection, the mice were treated with LPS. After that, these peptides were tested in vivo using intraperitoneal administration. Then, TNF-α level in whole brain lysate were measured by ELISA method. In the brain tissue, ex vivo TNF-α level were also decreased after LPS stimulation as compared with LPS control (*p* = 0.030 and *p* < 0.001). A short peptide (11 amino acid residues) VIPER of vaccinia virus A46 protein on both murine (iBMDM) and human cells (THP-1 and PBMC) in vitro at concentrations significantly decreased TNF-α production after LPS-stimulation (*E. coli*). Inhibiting resulted from the blocking interaction of TLR4 with Mal (TIRAP) and TRAM adaptor proteins [173]. Epta-peptides derived from BB-loop region of Toll/IL-1 receptor (TIR) domain also inhibited homodimerization of MyD88 TIR domains in vitro [174]. TIR-signaling modulation has been described in a review [175]. Therapeutic targets belonging to the TLR4 signaling pathway are discussed by Roy et al. [176]. It was concluded that disrupting the TLR4/ NF-κB pathway is a promising strategy to treat inflammatory disorders but it should not be blocked for long time because of its huge role in the host immune response.

## 6. Discussion and Conclusions

The TLR4 antagonists described in this review are synthetic or natural molecules belonging to different classes of compounds, from glycolipids to chalcone derivatives, to terpenoids, to peptides. 

These molecules have diverse modes of action, blocking the TLR4 signal at various stages and binding to their targets non-covalently as well as covalently. The exact targets of some TLR signaling pathway blockers are not clear yet and the hypotheses generated by docking studies need experimental validation. Many compounds that decreased pro-inflammatory cytokines (TNF-α, IL-6, IL-1β, IFN-γ) concentration after LPS stimulation in cell-based assays were also active in vivo on murine model, improving survival and decreasing the cytokine levels.

Among all TLR4 antagonists so far developed, only TAK-242 and Eritoran reached clinical trials, but unfortunately failed to pass them and were not active in improving patients survival [124,129,130,131,132]. At phase 3 of clinical trials, Eritoran was not active in improving survival also for sepsis patients infected by Gram-negative flora (21.9 vs 22.3%, *p* = 0.89), although for this subgroup a positive effect was expected. The negative results in clinic were in contrast with successful applications of TLR4 antagonists in sepsis therapy in animal models. This “translational gap” is well known in a series of other drugs, in the case of sepsis it could derive from the difference in TLR4 signaling and in the expression of inflammatory pathway genes in humans and in mice.

In a comparative review written by Vaure and Liu [177], the expression patterns of TLR4 in different tissues and mammal species were analyzed, and the relative sensibility of various animal species to LPS also considered. When compared to humans, mice are less susceptible to LPS, with the threshold dose required for minimal physiological changes is 0.5 mg/kg intraperitoneally for mice [178] (BALB/c strain) vs 1–5 ng/kg intravenously for humans [179]. This is a substantial difference even considering the diverse susceptibility across the mouse strains. One of the possible reasons for that is the difference in cytokine production patterns [177].

It is also known that after LPS stimulation in mouse neutrophils and peritoneal macrophages, a decrease in TLR4 expression occurs [180], in contrast, TLR4 expression in human monocytes is unaltered at least after low doses of lipopolysaccharide [181]. Cytokine production and NF-kB DNA-binding activity are also suppressed in mice [182]. Moreover, several mRNA isoforms of TLR4 in mice are known [183]. A notable one is smTLR4 (soluble mouse TLR4). This splicing variant consists of 122 amino acid residues, is not membrane-bound, and contains a part of the TLR4 extracellular domain responsible for the interaction with LPS. One possible reason can be the inhibition of TLR4/MD-2 interaction since MD-2 is known to be essential in mediating the LPS response. smTLR4 can therefore be an endogenous TLR4-antagonist acting on the early stages of TLR4 signaling. There is not an equivalent of such TLR4 isoform in humans.

It is also important to consider the similarity between animal sepsis models and human sepsis. As stated before, the CLP model mimics human sepsis the most but rarely was used in the studies reported in this review. The LPS injection model was often employed, which reproduces septic shock rather than sepsis. So for further development of TLR4-signaling modulators, their testing on CLP sepsis model is also recommended.

Taken together, these factors underline the great differences between murine and human innate immune response to LPS and explain, at least in part, the clinical gap in the case of sepsis.

Because of the complexity of innate immunity regulation, and also because of the redundancy of pathways involved in TLR-triggered cytokine production, in the future combined therapies could be investigated and tested in clinic.

One possibility could be to combine TLR4 antagonists with specific antibacterial agents.

Wang et al. [111] have studied the effect of the TLR4 antagonist artemisinin alone and in combination with antibiotics on the mice survival after administration of live *E. coli* bacteria as a source of LPS. When ampicillin sodium or a 2:1 mixture of ampicillin sodium/sulbactam sodium were co-administrated to the bacterium, the survival increased to 33% and 67%, respectively. When administering antibiotics alone, a 0% and 33% survival increase was obtained. Conversely, when administrating Artemisin alone, no protection was obtained after the injection of live *E. coli* bacteria.

Another case when TLR4 antagonist was given together with antimicrobial substance was described by Shirey and co-workers [51], albeit the disease was influenza and not sepsis. Applied together with Tamiflu™ (oseltamivir), Eritoran significantly improved the survival of C57BL/6J mice as compared with the group given oseltamivir alone, and protected them from lethal re-infection. 

A second possibility is the combination of blockers of different TLRs. In a recent successful example, anti-TLR2 and anti-TLR4 monoclonal antibodies were combined [184]. When metronidazole and ceftriaxone have been co-administrated with TLR4-targeting immunoglobulins, the survival increased more than twice when comparing with anti-TLR4 antibodies alone.

Concerning pathologies related to PAMP/TLR4 activation, in particular in the case of acute sepsis and septic shock, one can therefore conclude that, despite promising results on animal models, the clinical trials based on a single TLR4 antagonist has not led so far to a significant improvement in survival. We propose here combination therapies as a new strategy to overcome the limitations discussed above.

The use of TLR4 modulators in pathologies caused by TLR4 activation by endogenous DAMPs, however, is still at a very early stage and promising results have been obtained at a preclinical stage. Some small-molecular TLR4 blockers displayed a lack of toxicity and were efficient in blocking DAMP/TLR4 signaling and cytokine production in an array of inflammatory and autoimmune diseases [185,186,187]. These comprise of neuropathic pain, ALS, rheumatoid arthritis, vascular inflammation, atherosclerosis, and other pathologies reviewed here.

We are therefore convinced that some modern day chronic inflammations and autoimmune diseases that still lack specific pharmacological treatment could be efficiently targeted by new drugs interfering with TLR(4) activation and signaling.

## Figures and Tables

**Table 1:** Results of the phase II and III clinical trials for TLR4-antagonists.

Substance name	Mechanism of action	Clinical trial, design and results	Reference
TAK-242 (Resatorvid)	Binds covalently to Cys747 of TLR4-TIR domain and blocks TLR4/TIRAP and TLR4/TRAM interactions	Randomized, double-blind, placebo-controlled phase 2 trial (274 patients). Failed to suppress cytokine levels in patients with sepsis and shock or respiratory failure.	[124]
Eritoran (E5564)	Lipid A mimic, binds to MD-2	Phase 2, double-blind, placebo-controlled, ascending-dose study (152 patients). Eritoran was well tolerated but did not attenuated systemic inflammation significantly.	[127]
		Randomized, double-blind, placebo-controlled, multinational phase 3 trial (1961 patient). Treatment did not improve 28-day survival.	[131]
		Prospective, randomized, double-blind, placebo-controlled, multicenter, ascending-dose phase II trial (293 patients). Eritoran was well tolerated but the mortality was not decreased significantly.	[132]
